# Prevalence, patterns, and determinants of breastfeeding cessation among mothers of children under 24 months in Uganda

**DOI:** 10.1186/s12889-024-19028-1

**Published:** 2024-06-05

**Authors:** Florence Nakaggwa, Derrick Kimuli, Norah Namuwenge, Rebecca N. Nsubuga, Hellen Nayebare, Louis Kaboine, Immaculate Baseka, Kenneth Kasule, Sheila Nyakwezi, Solome Sevume, Norbert Mubiru, Barbara Amuron, Daraus Bukenya

**Affiliations:** 1 United States Agency for International Development, Strategic Information Technical Support (SITES) Activity, Social & Scientific Systems, Inc, DLH Holdings company, Kampala, Uganda; 2 The United States Agency for International Development Uganda, US Mission Compound-South Wing, Kampala, Uganda

**Keywords:** Breastfeeding cessation, LQAS, Complementary feeding

## Abstract

**Background:**

Breastfeeding duration is a critical component of infant and child nutrition, providing immediate and long-term benefits to both children and their mothers. This study uses data from the lot quality assurance sampling (LQAS) survey to examine the prevalence, patterns, and determinants of breastfeeding cessation in Uganda.

**Methods:**

This study was a secondary analysis of data collected by the cross-sectional LQAS surveys in 2021 and 2022 covering 77 districts in Uganda. The LQAS survey methodology employs a systematic sampling approach to assess whether predefined quality standards are met within specific subgroups of a population. The study employed spatial analysis, bivariate analysis and logistic regression, both with and without clustering, to explore associations between independent variables and breastfeeding cessation. Unadjusted and adjusted odds ratios with 95% confidence intervals were calculated. Statistical significance was set at 5%.

**Results:**

Overall, the study analysed 26,377 records of mothers with children under 24 months old. The mothers’ mean age was 27.9 years while that of their children was 11.0 months. While the general breastfeeding cessation rate was 17.7%, cessation was highest (49.7%) among mothers of children 18-23 months. Factors associated with increased odds of breastfeeding cessation included older child’s age, older mother’s age, using modern family planning, being pregnant and having an unknown pregnancy status. Lower odds of breastfeeding cessation were observed among mothers who; were married, lived in larger households, lived in rural residences, whose children received vitamin A supplementation and among all other regions compared to Kampala.

**Conclusion:**

One in five mothers cessed breastfeeding before their child reached 2 years, with a significant increase in cessation odds among mothers of older children. These findings underscore the importance of interventions to promote breastfeeding continuation and adequate nutrition for non-breastfed infants, particularly in regions with high cessation rates.

## Background

Breastfeeding is a critical component of infant and child nutrition, providing immediate and long-term benefits to both children and their mothers [1, 2]. For this reason, the World Health Organization (WHO) and the United Nations Children’s Fund (UNICEF) recommend that after 6 months, children should receive complementary foods with continued breastfeeding up to 2 years of age or beyond [3]. Breastmilk continues to meet up to 50% of a child’s energy needs between 6 and 12 months, and more than 30% between 12 and 24 months [1, 4]. Moreover, extended breastfeeding continues to offer essential immunological support which holds the potential to decrease morbidity and mortality among infants and young children [5]. On a global scale, while breastfeeding rates are high, disparities in rates are evident both between and within countries [3]. On the other hand, sustained breastfeeding of children up to 2 years exhibits lower rates and similarly displays variations both between and within countries [3]. The observed rates of breastfeeding are partly attributed to the presence or absence of enabling environments and policies that protect breastfeeding [6, 7]. This is compounded by poor community and social support structures [4] making it a compelling case for the need to increase awareness to raise breastfeeding rates from birth through two years [3].

In Sub-Saharan Africa (SSA), addressing child malnutrition remains a significant challenge within the public health domain [8]. Among the pivotal interventions to counteract malnutrition, the duration of breastfeeding holds exceptional importance [3]. Remarkable progress has been achieved in Uganda towards reducing child malnutrition, as evidenced by substantial decreases in stunting (from 45 to 29%) and underweight (from 18 to 11%) between 2000 and 2016 [9]. Despite this advancement, a noteworthy decline in the percentage of mothers continuing breastfeeding was observed shortly after the 12-month mark. Additionally, a concern persists regarding the proportion of children under 2 years who are still breastfeeding by the 24-month milestone [9]. Previous studies in Uganda have explored breastfeeding cessation in the context of human immunodeficiency virus (HIV) [10, 11]. The present national-level findings are dated, spanning over 7 years. Moreover, these findings lack the provision of additional insights into the determinants of breastfeeding cessation or district-based estimates [9]. According to UNICEF, the factors influencing maternal and child nutrition are often multifaceted, encompassing immediate, underlying, and enabling determinants [12]. The present study harnesses the lot quality assurance sampling survey (LQAS) data to highlight determinants of breastfeeding cessation. This study partly responds to UNICEF’s call for increased awareness to promote prolonged breastfeeding duration [3, 12]. It also addresses similar initiatives like the National Information Platforms for Nutrition in Uganda, which aim to improve analysis of existing data to inform strategic decisions to prevent malnutrition [13].

## Methods

### Dataset used

The study used the 2021 and 2022 LQAS survey datasets. The LQAS is a large-scale survey that provides an accurate measure of the coverage of service system quality at an aggregate level, such as at the district or regional level [14, 15]. It employs a small sample size to make binary decisions about the quality of individual units within distinct categories or areas. The method is designed to minimize costs and resources by making localized assessments rather than comprehensive evaluations. LQAS is particularly useful in situations where resources are limited and quick decisions are needed [14]. It involves the subdivision of a population into smaller units, often referred to as “lots,” which can encompass geographical regions, communities, or other defined subgroups. Each of these lots is categorized as either “acceptable” or “unacceptable,” contingent upon whether specific criteria or thresholds are met. In contrast to alternative rapid assessment methodologies, the compilation of outcomes from diverse lots contributes to the estimation of the overall coverage.

Each district underwent division into 5 to 7 lots (referred to as supervision areas) based on established criteria such as administrative boundaries and population attributes. The study employed the probability proportionate to size sampling technique, selecting either 19 or 24 villages from each designated lot. At the village level, the reference household was determined through a straightforward random sampling procedure. The initial interview was conducted with the nearest household to the reference point, if respondents meeting the criteria were available. In instances where they were not, subsequent households were considered until the survey was concluded. For respondents within households, selection was accomplished through simple random sampling when multiple categories or respondents within a category were present. More information about the survey and its routine application in Uganda can be found here [15–21]. The surveys covered 77 districts in Uganda and elicited responses from 26,377 mothers with children under 24 months of age among other respondents. For this study, a subset of the dataset was used. Data from mothers of children below 24 months was used for the analysis. Variables including participants background and breastfeeding history was abstracted and used for this analysis maintaining the cross-sectional design.

### Study variables and measurements

The study’s dependent variable was breastfeeding cessation, defined as the point at which a child younger than 24 months stopped receiving breastmilk. This variable was binary: mothers of children under 24 months were classified as either still breastfeeding or having stopped breastfeeding. This classification was based on the survey question, *“Are you currently breastfeeding this child?”* to which a *yes* or *no* response was given. To select independent variables, the study used variables from the survey that aligned with the UNICEF Conceptual Framework on Maternal and Child Nutrition [12]. According to this framework, the factors influencing maternal and child nutrition are multifaceted, encompassing immediate, underlying, and enabling determinants [12, 22]. Based on its depiction, the study chose the following as independent variables: age of the child and mother, child’s sex, mother’s marital status, highest level of education, geographic location characteristics, household size, antenatal care attendance, place of delivery, use of modern contraceptives, current pregnancy status, and mother’s diet (specifically whether she consumed food from at least three different food groups the day before the survey).

### Statistical analysis

Participant baseline and reproductive health characteristics were described using frequencies and percentages or means and corresponding standard deviations. In the bivariate analysis, categorical data were summarized using frequencies and percentages, while continuous data were summarized using means and standard deviations. The data were compared for disparities in the outcome through the Chi-square test for categorical variables and the t-test for continuous variables. Initially, at multivariate analysis, a multilevel logistic regression approach was employed, incorporating clustering at both the regional and district levels [23]. The goal was to explore differences in breastfeeding cessation between and within groups (regions and districts). The analysis grouped similar districts to facilitate the identification of distinct segments and anomalies to enable data exploration and extraction of meaningful insights. However, in the final analysis, the model without clustering was preferred after careful consideration of the estimates and corresponding standard errors.

The results revealed minimal disparities between the clustered and non-clustered models, indicating that the choice of the model had negligible impact on parameter estimates or their precision. In line with the principle of parsimony, the simpler model was the preferred option, adhering to the well-known Occam’s razor principle [24]. Unadjusted odds ratios (uORs) and adjusted odds ratios (aORs) were calculated alongside corresponding 95% confidence intervals (CI) as part of the analysis. Variables with a p-value below 0.05 were regarded as statistically significant. This selection ensured that the model’s complexity remained proportional to the data and research question, enhancing the clarity, transparency, and robustness of our findings. As such, the study confidently proceeded with the model without clustering, aligning with established statistical practices and promoting the interpretability of the results. The analysis was performed utilizing STATA version 17 [25]. Spatial analysis was an additional integral component of this study, enabling the exploration of geographic patterns and relationships within the dataset. To accomplish this, Quantum Geographic Information System (QGIS), an open-source GIS software, was employed. QGIS provides a versatile platform for performing various spatial analyses, ranging from basic geoprocessing tasks to more advanced geospatial modelling [26].

## Results

### Prevalence of breastfeeding cessation

**Fig. 1:**
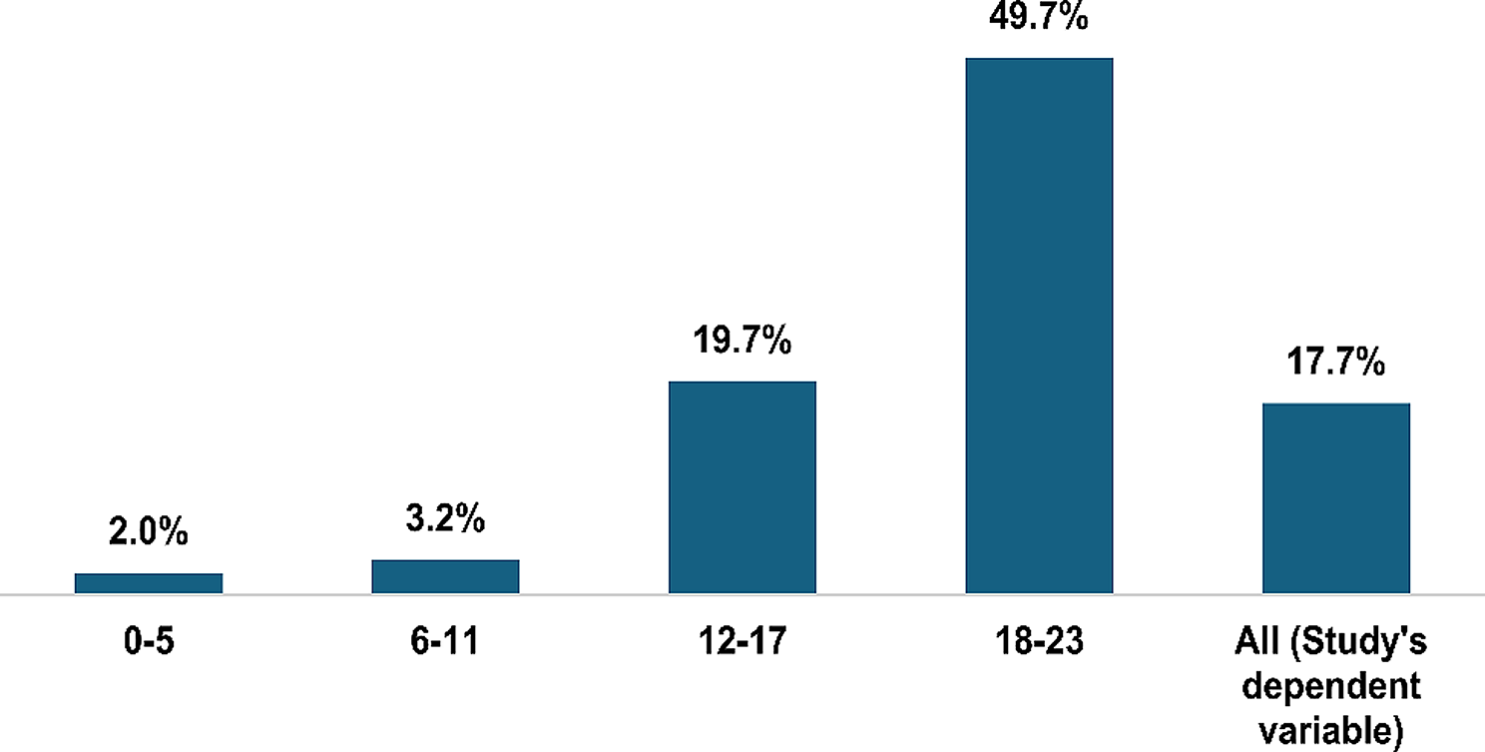
Cessation of breastfeeding by child’s age group

**Fig. 2:**
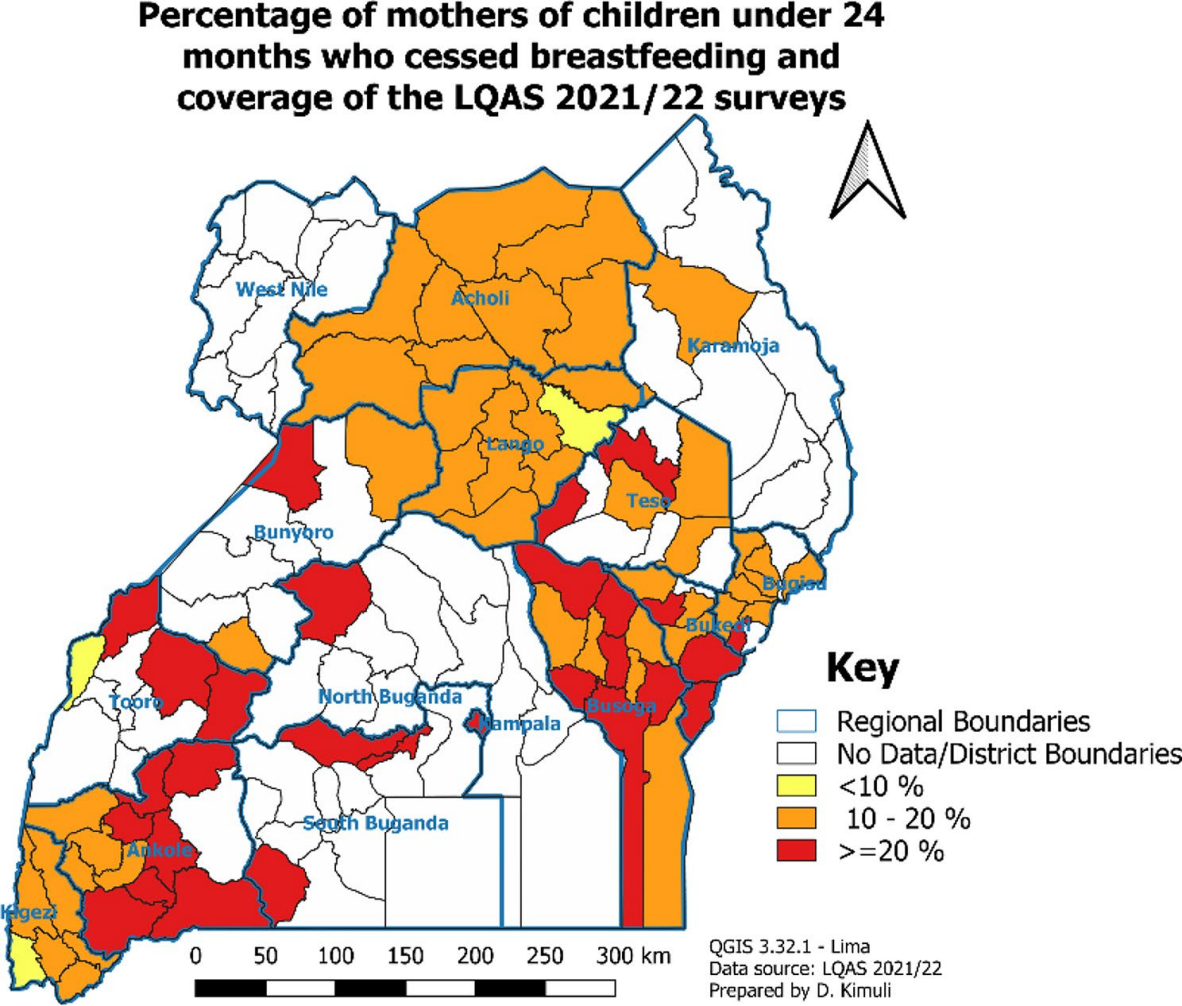
Cessation of breastfeeding before 24 months by district

In total, approximately 17.7% (95% CI 17.3–18.2) of mothers had cessed breastfeeding their children under the age of 2. Within age groups, the rates of breastfeeding cessation were highest among mothers of children aged 12–17 months and 18–23 months at 19.7% and 49.7% respectively. Also, the spatial analysis showed that the prevalence of cessation of breastfeeding differed by district, 28 of the 76 districts had an overall cessation rate of more than 20%. Figures 1 and 2 below show the prevalence of cessation of breastfeeding. Further details are also presented in Table 1.

### Participant characteristics and the prevalence of breastfeeding cessation of children under 24 months

Table 1 presents an overview of the participants’ sociodemographic characteristics concerning breastfeeding cessation. The dataset encompassed records from 26,377 mothers with children under 24 months of age. The average household size was 5.3 (standard deviation [SD] ± 2.4). The mean age of the children was 11.0 months (SD ± 6.8) [Median = 11, Interquartile range = 5–17], with a slight majority being female (51.2%). The mothers’ average age was 27.9 years (SD ± 37.6), and a significant portion was above 30 years old (33.1%). Most mothers were married (93.2%), had completed primary education (69.2%), lived in households with less than the mean household size (62.7%), and resided in rural areas of Uganda (80.1%). Significant statistical differences (*p* < 0.05) were observed in the rates of breastfeeding cessation based on marital status, mother’s age, educational attainment, mean household size, and place of residence. Breastfeeding cessation was more prevalent among mothers who were divorced, separated, or widowed (24.7%, *p* < 0.001), aged 30 years and older (19.9%, *p* < 0.001), with secondary education (19.4%, *p* = 0.001), living in households smaller than the mean household size (18.1%, *p* = 0.046), and residing in urban areas (18.9%, *p* = 0.011).

**Table 1:** Bivariate analysis of participant characteristics and breastfeeding cessation of children under 24 months

Variables	Frequency N = 26,377	Ceased breastfeeding before 24 months		*p*-value
		No. n = 21,706	Yes, n = 4,671	
Child age				< 0.001*
0–5 months	7,246 (27.5)	7,103 (98.0)	143 (2.0)	
6–11 months	5,949 (22.6)	5,761 (96.8)	188 (3.2)	
12–17 months	7,362 (27.9)	5,912 (80.3)	1,450 (19.7)	
18–23 months	5,814 (22.1)	2,926 (50.3)	2,888 (49.7)	
Child sex				0.543
Male	12,880 (48.8)	10,618 (82.4)	2,262 (17.6)	
Female	13,497 (51.2)	11,088 (82.2)	2,409 (17.8)	
Mother marital status				< 0.001*
Never married	645 (2.5)	487 (75.5)	158 (24.5)	
Married	24,591 (93.2)	20,360 (82.8)	4,231 (17.2)	
Divorced/separated/widowed	1,141 (4.3)	859 (75.3)	282 (24.7)	
Mother age				< 0.001*
10–19	2,563 (9.7)	2,288 (89.3)	275 (10.7)	
20–24	8,389 (31.8)	6,965 (83.0)	1,424 (17.0)	
25–29	6,686 (25.3)	5,455 (81.6)	1,231 (18.4)	
30+	8,744 (33.1)	6,998 (80.1)	1,741 (19.9)	
Mother education level				0.001*
None	1,628 (6.2)	1,318 (81.0)	310 (19.0)	
Primary	18,251 (69.2)	15,107 (82.8)	3,144 (17.2)	
Secondary	4,892 (18.5)	3,943 (80.6)	949 (19.4)	
Above secondary	1,606 (6.1)	1,338 (83.3)	268 (16.7)	
Household size (hhs)				0.046*
<=mean hhs	16,541 (62.7)	13,552 (81.7)	2,989 (18.1)	
>mean hhs	9,836 (37.3)	8,154 (82.9)	1,682 (17.1)	
Residence				0.011*
Urban	5,239 (19.9)	4,248 (81.1)	991 (18.9)	
Rural	21,138 (80.1)	17,458 (82.6)	3,680 (17.4)	

N = Overall Total, n = subtotal, *Denotes statistical significance at *p* < 0.05, Percentages shown in brackets

#### Reproductive health, feeding patterns, and prevalence of breastfeeding cessation of children under 24 months

Table 2 provides a comprehensive bivariate analysis of reproductive health factors, feeding patterns, and breastfeeding cessation. The participants’ mean attendance at ANC was 4.7 times (SD 1.7), with a vast majority having engaged in ANC (97.7%). Notably, 31.5% attended ANC in the 3rd month of pregnancy, and 52.6% attended within the first trimester. Also a significant majority delivered their child at a health facility (86.3%), received infant feeding counselling (77.3%), received vitamin A supplementation for their child (59.7%), refrained from introducing solid foods before 6 months (78.5%), were not members of a mother care group (94.1%), ate at least three food groups the day preceding the survey (78.8%), did not employ modern family planning (FP) methods (75.0%), and were not pregnant during the survey (91.9%).

Statistically significant disparities in the rate of breastfeeding cessation were observed with numerous factors, including ANC attendance, the timing of the 1st ANC visit, frequency of ANC visits, ANC attendance within the first trimester, place of childbirth, receipt of child’s vitamin A supplementation, maternal diet, use of modern FP methods, and pregnancy status. Mothers who, did not attend ANC (28.5%, *p* = 0.001), initiated their first ANC visit at 4 months (18.3%, *p* = 0.035), missed ANC within the first trimester (17.9%, *p* = 0.043), gave birth at home (19.2%, *p* = 0.014), were uncertain about their child’s vitamin A supplementation status (20.8%, *p* < 0.001), maintained a good maternal diet (19.4%, *p* < 0.001), employed modern FP methods (28.5%, *p* < 0.001), and were currently pregnant (62.3%, *p* < 0.001) cessed to breastfeed.

**Table 2:** Bivariate analysis of reproductive health, feeding patterns and breastfeeding cessation of children under 24 months

Variables	Frequency N = 26,377	Ceased breastfeeding		*p*-value
		No, n = 21,706	Yes, n = 4,671	
ANC Attendance				0.001*
No	613 (2.3)	438 (71.5)	175 (28.5)	
Yes	25,764 (97.7)	21,268 (82.5)	4,496 (17.5)	
Months at 1st ANC				0.035*
1	1,564 (6.1)	1,315 (84.1)	249 (15.9)	
2	3,870 (15.0)	3,242 (83.8)	628 (16.2)	
3	8,110 (31.5)	6,685 (82.4)	1,425 (17.6)	
4	6,544 (25.4)	5,343 (81.7)	1,201 (18.3)	
5	5,676 (22.0)	4,683 (82.5)	993 (17.5)	
ANC attendance times				0.001*
Mean (SD)	4.7 (1.7)	4.8 (1.7)	4.6 (1.7)	
ANC attendance in 1st trimester				0.043*
No	12,220 (47.4)	10,026 (82.1)	2,194 (17.9)	
Yes	13,544 (52.6)	11,242 (83.0)	2,302 (17.0)	
Delivery place				0.014*
Home/other	3,617 (13.7)	2,924 (80.8)	693 (19.2)	
Health facility	22,760 (86.3)	18,782 (82.5)	3,978 (17.5)	
Counselling on infant feeding				0.669
No	5,856 (22.7)	4,845 (82.7)	1,011 (17.3)	
Yes	19,908 (77.3)	16,423 (82.5)	3,485 (17.5)	
Vitamin A supplementation				< 0.001*
No	10,088 (38.3)	8,737 (86.6)	1,351 (13.4)	
Don’t know	528 (2.0)	418 (79.2)	110 (20.8)	
Yes	15,761 (59.7)	12,551 (79.6)	3,210 (20.4)	
Solid food introduced before 6				0.709
months	14,961 (78.5)	11,439 (76.5)	3,522 (23.5)	
No	4,093 (21.5)	3,118 (76.2)	975 (23.8)	
Yes				
Member mother care group				0.733
No	24,824 (94.1)	20,433 (82.3)	4,391 (17.7)	
Yes	1533 (5.9)	1,273 (82.0)	280 (18.0)	
Mother diet				< 0.001*
No	20778 (78.8)	17,192 (82.7)	3,586 (17.3)	
Yes	5599(21.2)	4,514 (80.6)	1,085 (19.4)	
Modern FP use				< 0.001*
No	19,788 (75.0)	16,992 (85.9)	2,796 (14.1)	
Yes	6589 (25.0)	4,714 (71.5)	1,875 (28.5)	
Currently pregnant				< 0.001*
No	23230 (91.9)	19,861 (85.5)	3,369 (14.5)	
Don’t know	514 (2.0)	374 (72.8)	140 (27.2)	
Yes	1522 (6.0)	574 (37.7)	948 (62.3)	

Abbreviations

ANC – Antenatal Care

SD-Standard deviation

FP – Family planning

*Percentages shown in brackets*

### Factors associated with breastfeeding cessation of children under 24 months

Table 3 presents a detailed multivariate analysis of factors linked to the cessation of breastfeeding. In the final model, ANC attendance in the first trimester, months at first ANC and ANC attendance times were excluded due to collinearity concerns, ensuring model convergence and appropriateness. The odds of breastfeeding cessation were as high as almost 7-fold and 28-fold with child’s age: 12–17 months (aOR 6.9, 95% CI 5.8–8.1, *p* < 0.001) and 18–23 months (aOR 28.33, 95% CI 24.0-33.5, *p* < 0.001), respectively. Similarly, the odds were higher by 20% among mothers who used modern FP methods (aOR 1.2, 95% CI 1.1–1.3, *p* < 0.001), 50% or 8 times higher among mothers who were either unaware of their pregnancy or currently pregnant (aOR 1.5, 95% CI 1.2-2.0, *p* < 0.001) and (aOR 8.0, 95% CI 6.9–9.3, *p* < 0.001) respectively.

Moreover, compared to younger mothers aged 10–19, the odds were higher by 30–50% based on maternal age brackets: 20–24 years (aOR 1.3, 95% CI 1.1–1.5, *p* = 0.004); 25–29 years (aOR 1.4, 95% CI 1.1–1.6, *p* = 0.001); and 30 years and above (aOR 1.5, 95% CI 1.2–1.8, *p* < 0.001). Additionally, compared to Kampala, the odds of breastfeeding cessation were lower in all but 2 regions (North and South central). Conversely, odds were lower by 40% for children of married mothers compared to unmarried mothers (aOR 0.6, 95% CI 0.4–0.7, *p* < 0.001), 10% for households exceeding the mean household size (aOR 0.9, 95% CI 0.8–0.9, *p* = 0.001), 10% among mothers residing in rural areas (aOR 0.9, 95% CI 0.8-1.0, *p* = 0.065), and 20% for mothers whose children had received vitamin A supplementation (aOR 0.8, 95% CI 0.7–0.9, *p* < 0.001).

**Table 3:** Factors associated with breastfeeding cessation of children under 24 months

Variables	Unadjusted OR (95% CI)	*p*-value	Adjusted OR (95% CI)	*p*-value
**ANC attendance in 1st trimeste r**□				
No	1			
Yes	0.9 (0.9-1.0)	0.043*		
**Months at 1st ANC**□				
1	1			
2	1.0 (0.9–1.2)	0.781		
3	1.1 (1.0-1.3)	0.114		
4	1.2 (1.0-1.4)	0.024		
5	1.1 (1.0-1.3)	0.144		
**ANC attendance times**				
Mean (SD)	0.9 (0.9-1)	< 0.001*		
Child age				
6–11 months	1		1	
12–17 months	7.5 (6.4–8.8)	< 0.001*	6.9 (5.8–8.1)	< 0.001*
18–23 months	30.2 (25.9–35.3)	< 0.001*	28.3 (24.0.-33.5)	< 0.001*
**Mother marital status**				
Never married	1		1	
Married	0.6 (0.5–0.8)	< 0.001*	0.6 (0.4–0.7)	< 0.001*
Divorced/separated/widowed	1.0 (0.8–1.3)	0.918	1.0 (0.8–1.4)	0.902
**Mother age**				
10–19	1		1	
20–24	1.7 (1.5-2.0)	< 0.001*	1.3 (1.1–1.5)	0.004*
25–29	1.9 (1.6–2.2)	< 0.001*	1.4 (1.1–1.6)	0.001*
30+	2.1 (1.8–2.4)	< 0.001*	1.5 (1.2–1.8)	< 0.001*
**Mother education level**				
None	1		1	
Primary	0.9 (0.8-1.0)	0.064	0.9 (0.8–1.1)	0.369
Secondary	1.0 (0.9–1.2)	0.752	1.1 (0.9–1.3)	0.303
Above secondary	0.9 (0.7-1.0)	0.081	0.8 (0.6-1.0)	0.128
**Household size (hhs)**				
<=mean hhs	1		1	
>mean hhs	0.9 (0.9-1.0)	0.046*	0.9 (0.8–0.9)	0.001*
**Residence**				
Urban	1		1	
Rural	0.9 (0.8-1.0)	0.011*	0.9 (0.8-1.0)	0.065
**ANC Attendance**				< 0.001*
No	1		1	
Yes	0.5 (0.4–0.6)	< 0.001*	0.6 (0.4–0.7)	
**Delivery place**				
Home/other	1		1	
Health facility	0.9 (0.8-1.0)	0.014	1.0 (0.9–1.1)	0.559
**Vitamin A supplementation**				
No	1		1	
Don’t know	1.7 (1.4–2.1)	< 0.001*	1.1 (0.8–1.4)	0.660
Yes	1.7 (1.5–1.8)	< 0.001*	0.8 (0.7–0.9)	< 0.001*
**Mother diet**				
No	1		1	
Yes	1.2 (1.1–1.2)	< 0.001*	1.0 (0.9–1.1)	0.610
**Modern FP use**				
No	1		1	
Yes	2.4 (2.3–2.6)	< 0.001*	1.2 (1.1–1.3)	< 0.001*
**Currently pregnant**				
No	1		1	
Don’t know	2.2 (1.8–2.7)	< 0.001*	1.5 (1.2-2.0)	< 0.001*
Yes	9.7 (8.7–10.9)	< 0.001*	8.0 (6.9–9.3)	< 0.001*
**Region**				
Kampala	1		1	
Acholi	0.5 (0.4–0.6)	< 0.001*	0.4 (0.3–0.6)	< 0.001*
Ankole	0.9 (0.8–1.1)	< 0.181	0.7 (0.5-1.0)	0.053
Bugisu	0.7 (0.6–0.9)	< 0.001*	0.5 (0.4–0.7)	< 0.001*
Bukedi	0.8 (0.7-1.0)	< 0.037*	0.7 (0.5-1.0)	0.034*
Bunyoro	0.7 (0.6–0.9)	0.006*	0.7 (0.5-1.0)	0.027*
Busoga	0.9 (0.7–1.1)	0.185	0.8 (0.6–1.1)	< 0.157
Kigezi	0.6 (0.5–0.7)	< 0.001*	0.4 (0.3–0.6	< 0.001*
Lango	0.5 (0.4–0.6)	< 0.001*	0.4 (0.3–0.5)	< 0.001*
North Central	1.3 (1.0-1.7)	< 0.021*	1.4 (0.9–2.1)	0.272
South Central	1.2 (1.0-1.5)	0.046*	1.2 (0.9–1.7)	0.272
Tooro	0.8 (0.6–0.9)	< 0.009*	0.6 (0.4–0.9)	0.006*
Karamoja	0.5 (0.3–0.7)	< 0.001*	0.3 (0.2–0.6)	< 0.001*
Teso	0.9 (0.7–1.1)	< 0.175	0.8 (0.6–1.2)	0.272

* denotes statistical significance at *p* = 0.05; 0–5 months group omitted; □ variable omitted from adjusted model due to collinearity

Abbreviations

ANC – Antenatal Care

hhs – Household size

SD – Standard deviation

FP – Family planning

SD – Standard deviation

## Discussion

The study findings revealed that about one in five mothers with children under 2 years old cessed breastfeeding before the child reached 2 years of age. Half of the mothers of children aged 18 - 23 years had cessed breastfeeding. The supplementary analysis also revealed an 80% higher the odds of cessation at the 6-month age mark. Moreover, the odds of cessation were higher among mothers who utilized modern FP methods, those who were pregnant or unaware of their pregnancy status and older maternal age. Conversely, the odds of cessation were lower among married mothers, those in households larger than the mean household size, mothers residing in rural areas or regions other than Kampala and those whose children had received vitamin A supplementation.

Close to 20% of the children were cessed from breastfeeding before attaining the age of 2 years. Notably, the likelihood of cessation escalated progressively by child’s age group. These observations mirror the findings of the 2016 Uganda Demographic and Health Survey (UDHS), which indicated that approximately 50% of mothers cessed breastfeeding by their children’s 20-month mark [9]. Hence, there possibly hasn’t been a discernible change in the practices regarding breastfeeding cessation in Uganda. The study highlighted an increased risk of breastfeeding cessation by mothers with children in older child age groups. This could be for multiple reasons, the end of maternity leave and the need to return to work [27–29], the introduction of complementary feeding [3] or the desire/practice to end breastfeeding early. This may potentially lead to improper assumptions about complementary food sufficiency. However, the high early breastfeeding cessation rate is exacerbated by the reality that merely around 15% of children the age group receive an adequate diet [9]. In tandem, these increase vulnerabilities to infections, malnutrition, and various health complications among children in Uganda [1–3]. The findings advocate for interventions aimed improving nutrition for non-breastfed infants [30] and routine monitoring of breastfeeding cessation by the health management information system [31]. Currently it can only be established through surveys such as the LQAS [17, 18] and UDHS [9].

An elevated likelihood of breastfeeding cessation was evident in mothers employing modern FP methods. Some studies found that the reliance on hormonal components potentially hindered breastfeeding by affecting milk production [32–34]. For the present study, the possible reason for this observation requires further investigation. Moreover, a recent study in Uganda found a puzzling connection between breastfeeding and postpartum FP: the odds of FP use were more among breastfeeding mothers, yet among non-FP users, most cited breastfeeding as their reason for not using FP [19]. Also, that the present study found higher odds of breastfeeding cessation with being pregnant mirrors the findings in Lebanon. For the Lebanon study, well-planned pregnancies were linked to higher odds of breastfeeding [35]. Modern FP methods play a pivotal role in effective pregnancy planning, so the study’s findings that FP use and pregnancy were associated with more odds of breastfeeding cessation warrant further investigation.

One surprising finding of this study was that older maternal age was a risk factor for breastfeeding cessation as this is contrary to other research [36–38]. In the present study, teenage mothers were more likely to breastfeed longer. A possible explanation is the high unemployment rates among teenage mothers which can reduce the possibility of employment-related breastfeeding cessation [39–41]. Unemployment can also lead to financial constraints which could limit the ability of teenage mothers to provide alternative comprehensive foods [42]. This could make prolonged breastfeeding one of the ways to ensure that the nutrition needs of their children are met. Also, this study observed regional and district disparities in the cessation of breastfeeding like what has been observed elsewhere [3]. This could be partly attributed to socioeconomic differences as much as cultural diversity [43, 44]. Notably, lower odds of cessation of breastfeeding were observed among mothers not living in Kampala (the country’s capital city). Moreover, overall, lower odds were observed among mothers living in rural areas. These observations are the same and are like those made in Ethiopia [35]. Women in the city or urban areas have more work demands or access to breastfeeding substitutes among other reasons which may compromise the duration of breastfeeding [39, 45]. Many workplaces lack essential breastfeeding support structures which hinders successful breastfeeding experiences [39–41].

Moreover, the odds of cessation were lower among married mothers and mothers in households larger than the mean household size. Marriage provides an opportunity for financial, social, and emotional support from spouses or relatives [46]. This can create part of the social support structures necessary to ensure proper childhood nutrition [4]. Although larger households may provide a risk for food insecurity [47], they can provide an opportunity for additional social support structures as the mother may be afforded time for childcare while other household members support with extra household duties. Therefore, households with higher sizes could leverage this opportunity to promote prolonged breastfeeding. Also, the study found that mothers whose children had received Vitamin A supplementation were less likely to cease breastfeeding. Vitamin A supplementation is given by community health workers or health workers at the facility. This may mean that such mothers had an opportunity to receive additional information on breastfeeding, unlike their counterparts [48]. Without Vitamin A supplementation among children who have stopped breastfeeding, both the benefits of prolonged breastfeeding [1, 4, 5] and Vitamin A supplementation [49–51] are missed. This is further exacerbated by an inadequate diet in this age group where the majority do not receive the minimum acceptable diet [9].

The present study examines breastfeeding cessation in Uganda using the LQAS datasets which, although not conventionally used for such investigations, offers a unique and current perspective. Remarkably, the study’s robustness lies in its expansive sample size, which ensures comprehensive representation from diverse districts and regions, thus bolstering the study’s applicability to a wider context. Nonetheless, the study does encounter certain limitations that warrant consideration while interpreting or employing its outcomes. A notable constraint resides in the study’s foundation upon self-reported data garnered from the LQAS survey. This reliance introduces a potential for bias, as respondents may not consistently convey their experiences with precision [52, 53]. To its benefit, the interval was only two years which could have increased the probability of accurate recall. Moreover, the LQAS data upon which the study is based covered 77 of the 135 districts in Uganda, representing about 57% of the country, which although localized, may raise pertinent questions regarding the extrapolation of findings to the entire country. However, similar studies in the country used much smaller sample sizes or were limited in scope being health facility-based or focused on the cessation of breastfeeding in the context of HIV [10, 11, 43, 44]. Consequently, this study’s findings provide a holistic and complementary understanding of breastfeeding practices beyond the constraints of previous research methodologies.

## Conclusion

The study examined the prevalence, patterns, and determinants of breastfeeding cessation among mothers of children under 24 months in Uganda. The findings show that one in five mothers cessed breastfeeding before their child reached 2 years of age, however, half of mothers with children 18 - 23 months had cessed breastfeeding. This implies that prolonged breastfeeding remains low in Uganda. The use of modern FP methods, pregnancy, older maternal age, and urban residence were associated with higher cessation odds, while marriage, larger household size, rural residence, and Vitamin A supplementation for children were linked to lower odds. While echoing findings from the 2016 UDHS [9], the study emphasizes the need for interventions on breastfeeding duration, particularly given the limited access to sufficiently nutritious diets for children in Uganda. Moreover, the findings call for more strategies for adequate nutrition for non-breastfed infants, particularly in regions with higher cessation rates.

## Data Availability

The data that support the findings of this study are available upon request from Social & Scientific Systems, Inc., a DLH Holdings Company. In compliance with our institutional data-sharing policy and due to the sensitive nature of the data, we are unable to publicly provide the data as part of the manuscript submission. To obtain the data from Social & Scientific Systems, Inc., a DLH Holdings Company, interested researchers should contact Daraus Bukenya (Dr.) at Daraus.Bukenya@dlhcorp.com, Barbara Amuron (Dr.) at Barbara.Amuron@dlhcorp.com or the DLH Institutional Review Board at IRBHelp@dlhcorp.com. If researchers prefer to request data from specific districts, they may reach out to the corresponding district offices through the Ministry of Local Government at ps@molg.go.ug. Requests for data from both Social & Scientific Systems, Inc., A DLH Holdings Company will be subject to review by the IRB Review Team, ensuring adherence to ethical and legal considerations.

## Abbreviations

ANC: Antenatal care
aOR: Adjusted odds ratios
FP: Family planning
Hhs: Household size
HIV: Human immunodeficiency virus
LQAS: Lot quality assurance sampling survey
PPFP: Postpartum family planning
QGIS: Quantum Geographic Information System
SSA: Sub-Saharan Africa
SD: Standard deviation
uOR: Unadjusted odds ratios
UDHS: Uganda Demographic and Health Survey
UNICEF: The United Nations Children’s Fund
WHO: World Health Organization
